# Alternative reproductive tactics and inverse size-assortment in a high-density fish spawning aggregation

**DOI:** 10.1186/s12898-017-0120-5

**Published:** 2017-02-28

**Authors:** Rucha Karkarey, Amod Zambre, Kavita Isvaran, Rohan Arthur

**Affiliations:** 1 Oceans and Coasts Program, Nature Conservation Foundation, 3076/5, 4th Cross, Gokulam Park, Mysore, Karnataka 570002 India; 2 Manipal University, Manipal, Karnataka 576104 India; 3 Post-graduate Programme in Wildlife Biology and Conservation, National Centre for Biological Sciences–Wildlife Conservation Society, Bangalore, Karnataka India; 4 Centre for Ecological Sciences, Indian Institute of Science, Bangalore, Karnataka India; 5 Centre d’Estudis Avançats de Blanes (CSIC), Blanes, Spain

**Keywords:** Spawning aggregation, High mating density, Alternative reproductive tactics, Shoal and pair courtship tactics, Inverse size-assortment, Squaretail grouper

## Abstract

**Background:**

At high densities, terrestrial and marine species often employ alternate reproductive tactics (ARTs) to maximize reproductive benefits. We describe ARTs in a high-density and unfished spawning aggregation of the squaretail grouper (*Plectropomus areolatus*) in Lakshadweep, India.

**Results:**

As previously reported for this species, territorial males engage in pair-courtship, which is associated with a pair-spawning tactic. Here, we document a previously unreported school-courtship tactic; where territorial males court multiple females in mid-water schools, which appears to culminate in a unique ‘school-spawning’ tactic. Courtship tactics were conditional on body size, local mate density and habitat, likely associated with changing trade-offs between potential mating opportunities and intra-sexual competition. Counter-intuitively, the aggregation showed a habitat-specific inverse size-assortment: large males courted small females on the reef slope while small males courted equal-sized or larger females on the shelf. These patterns remained stable across two years of observation at high, unfished densities.

**Conclusions:**

These unique density-dependent behaviours may disappear from this aggregation as overall densities decline due to increasing commercial fishing pressure, with potentially large consequences for demographics and fitness.

**Electronic supplementary material:**

The online version of this article (doi:10.1186/s12898-017-0120-5) contains supplementary material, which is available to authorized users.

## Significant statement

Mating successfully at high densities often requires species to employ unusual reproductive tactics. We report unique courtship behaviours in an unfished, high-density spawning aggregation of squaretail groupers (*Plectropomus areolatus*) that are potentially associated with alternative reproductive tactics (ARTs). Aggregating males are typically known to court females in small territories (pair courtship), which is often associated with a pair-spawning tactic. However, we also observed the largest males simultaneously courting several females in mid-water shoals – a unique, high-cost-high-benefit courtship tactic which appears to result in a novel school-spawning tactic. Counter-intuitively we observed an inverse size-assortment in individuals–large males courted smaller females and vice-a-versa, likely linked to different pay-offs with competitive ability and local mate density. These unique, high-density behaviours are threatened to be lost, with increasing commercial fishing pressures on the * P. areolatus* aggregation.

## Background

Ensuring reproductive success in competitive high-density populations often requires individuals to adopt innovative mating strategies. Reproductive strategies are strongly mediated by density—i.e. the number of potential mates (local mate density) as well as the overall population density [1, 2]. High local mate density in a population increases competition for mates. Under these circumstances, if a few individuals are able to monopolize mates, most others will have little success [3]. This skew in reproductive success often selects for multiple male and female phenotypes or alternative ways of acquiring reproductive benefits, commonly known as alternative reproductive tactics (ARTs, [4]).

Overall population densities may impact alternative reproductive strategies in a population in unpredictable ways [2]. For example, high-density conditions could result in significant density-dependent effects such as space limitation and the inability of competitors to fight off multiple intruders [1]. This may lead to a breakdown in mate monopoly [5], lowering the reproductive skew in a population and consequently suppressing the expression of ARTs [6]. However, increasing population density may trigger variations in mate choice [7, 8], which may serve to increase reproductive skew and select for costly, novel or elaborate ARTs [2, 9, 10]. Across multiple taxa, large male size is favoured, either through male-male competition or female choice, with little selection on female size (e.g. fish [11]; mammals [12, 13]). However, in some taxa [14–16], males also show a preference for large females, resulting in mating pairs where male and female sizes are positively correlated (‘size assortment’, 16). The overall population density can impact the strength of sexual selection on male and female traits through its effects on intra-sexual competition [17–20].

Animal mating aggregations lie at one extreme of the density spectrum, and can provide valuable insights in understanding size-selection and mating systems in high-density conditions. Fish spawning aggregations are ideal systems to study this relationship because several species spawn in spatially and temporally explicit aggregations that often attain very high densities [21, 22]. A rich body of literature dating back to Aristotle [23] has shown that fish have highly variable and flexible mating modes, ranging from pair-spawning and group-spawning tactics, demersal and broadcast spawning tactics, to gonochorism and hermaphroditism [19, 24–29]. In addition, fish show some of the strongest tendencies for positive size-assortment among animal taxa [16]. Fish mating systems can vary considerably between closely related species or even regional populations of the same species [30]. These differences are often context (habitat, local density) and condition (body size, age) dependent [19, 28, 31]. In the absence of adequate field data for many aggregating fish species, we often rely on generalisations of mating behaviour from closely-related species or populations from better-studied regions. Moreover, ‘pristine’ or unfished fish spawning aggregations are rare in the wild, and this is particularly true of large-bodied and commercially important marine fish species [32], impeding our understanding of how many species behave under natural high-density conditions.

Groupers (Teleostei: Epinephelidae: Epinephelini [33]) are large-bodied fish, ubiquitous to coral reefs. They are functionally important predators, and many species form high-density spawning aggregations [34]. Groupers possess complex mating systems with several sex-changing species [28, 35, 36]. Reproductive strategies and sex-change patterns in groupers can be strongly mediated by local mate density and overall population density [36, 37]. However, because groupers are highly prized food-fish [38], their spawning aggregations are heavily targeted by commercial fisheries [39]. Fishing can severely alter population density and the size-structure of a spawning aggregation [22, 32, 40, 41] potentially affecting the mating system. Unfished spawning aggregations, where they still persist, can therefore provide critical baselines and novel insights into grouper mating systems under rare, natural high-density conditions.

The squaretail grouper (*Plectropomus areolatus*) is a common plectropomid species found across the Indo-Pacific region. Previous work observed *P. areolatus* using a pair-spawning tactic where principally large males establish and defend territories at the aggregation site, which are then visited by gravid females [42, 43]. Males court females within their territories and this is associated with pair-spawning just above the male’s territory. In 2011, we documented an unfished, high-density aggregation of the squaretail grouper at a remote atoll in Lakshadweep, India. Our observations reveal an additional school-associated courtship tactic, distinct from earlier reports in the literature for this species. We describe this novel courtship tactic as school-courtship, and suggest that this leads to a unique school-spawning tactic in high-density *P. areolatus* spawning aggregations.

Few studies have described ARTs in grouper spawning aggregations [31, 36, 42, 43] and to our knowledge, no studies have evaluated ARTs in plectropomid species. Here, in addition to describing a unique spawning tactic, we examine and evaluate ARTs in an unfished, high-density squaretail grouper spawning aggregation over two years (2013 and 2014). Specifically, we evaluate (1) male and female preference for body size (size-assortment) in the two habitats (shelf and slope), by examining their relative spatial distributions. (2) The frequency of two distinct male courtship tactics in the two habitats, and describe how these potentially lead to two alternative spawning tactics and (3) the potential costs and benefits associated with the different courtship tactics.

## Methods

### Study area and site

The study was conducted in Bitra, a remote atoll in the northern Lakshadweep archipelago. The archipelago lies roughly 400 km off the state of Kerala, along the south-west coast of India. Bitra has a small island (0.105 km^2^ area), with a community of less than 200 people. The atoll encloses a large lagoon of 46.51 km^2^ surrounded by coral reefs.

Until recently, local fishing in Bitra and other atolls has been largely an artisanal enterprise, mainly targeting off-shore tuna stocks [44]. Our study was conducted in 2013 and 2014, prior to which there was relatively low reef fishing pressure in Bitra. During the course of our study there was a complete administrative ban on fishing activities on Bitra’s reefs during the aggregation period.

Due to the remote location of the island and associated logistical challenges, we were able to survey Bitra only opportunistically since 1998 (n = 6 years, 1998, 2011–2015) between the months of December–April. Based on these opportunistic surveys and local fishermen interviews, the study was conducted in the new moon of January (2013 and 2014), around peak aggregation densities.

In 2012, we demarcated the boundaries of the aggregation site based on the presence of territory-holding males, by surveying the area on SCUBA and snorkel. The area of the aggregation was estimated to be approximately 40,000 m^2^ comprising a contiguous stretch of reef separated by sand patches. The site can broadly be divided into two habitats, reef shelf and reef slope. The reef shelf starts at a depth of 6 m sloping gently to 11 m where it transitions to a steep reef slope. The reef shelf stretches nearly 170 m in breadth. The reef slope begins at a depth of 11 m descending sharply at an approximately 45° angle, to sand at 20 m. The reef shelf and slope at the aggregation site were very similar in terms of benthic coral structure, dominated by large *Porites* and *Diploastrea* boulders.

### Annual aggregation density

Across the Indo-Pacific, densities of *P. areolatus* aggregations peak either on the day of the new moon or full moon [45]. In Bitra, this species appears to spawn over the new moon (RK, RA, AZ, personal observations). We surveyed the aggregation annually for 5 days (2 days of waning crescent, new moon and 2 days of waxing crescent) during the new moon lunar phase in January/February of each year, based on our prior observations of the build-up of numbers and duration of the aggregation.

Sampling was focused in a core area of approximately 2500 m^2^, which covered 6.5% of the total aggregation area (40,000 m^2^). The densest part of the aggregation or the ‘core aggregation area’ [46] was defined as the area within which large female schools roved during the aggregation period [42]. In this core area, we established 5 permanent belt transects (50 m × 10 m, 2 slope and 3 shelf transects), following methodology in [47]. Transects were placed 10 m apart. Transects on the slope and shelf were placed parallel to one another with a minimum distance of 25 m between them. The vertical extent of the sampling area was approximately 5 m, based on movement of fish in the water column. We surveyed these transects every day over the 5 day period in 2013 and 2014, during low tide and compared new moon peak densities from sampling surveys conducted in 2013 (10th February) and 2014 (30th January). Transects were swum by two observers, and a mean of total count of individuals taken by each observer in a volume of 2500 m^3^ was used as transect density.

Mean (±SE) annual core density was estimated from transect densities (n = 10 transects, 5 transects × 2 years), for surveys conducted on peak days in 2013 (10th February) and 2014 (30th January).

### Male and female density distribution: size assortment

We used timed stationary point counts to compare male and female densities on slope and shelf habitats on peak aggregation days in 2013 (30th January) and 2014 (10th February). This additional sampling technique was used to document sex of individuals which was not included in the permanent transect surveys.

We randomly established 5–6 survey points in each habitat within the core aggregation area and sampled each point for a total of 5 min (total n = 23). At each point count we noted the abundance, size and sex of individuals within a cylinder of 5 m radius and 5 m height of the survey point (volume ~393 m^3^). On peak aggregation days (new moon days), we assumed that all individuals with distended bellies were females. We validated this assumption by opportunistically catching and (non-fatally) sexing 24 individuals on peak aggregation days (January 2012 and 2015). All individuals with distended bellies were found to be females (n = 11) and those with flat bellies were found to be males (n = 13). Of these, males and females had overlapping sizes: Male body size ranged between 40 and 74 cm, and female size ranged between 36 and 56 cm. Males and females were binned into fifteen centimetre size classes. We binned individuals post hoc to categorize males that overlapped in size with females and those which did not overlap in size with females. In previous studies [42, 43], males that overlapped in size with females were often found to be non-territorial and roving with female schools, while larger males held territories at the aggregation site. Males were thus classified as small (40–55) cm and large (56+ cm) to study differences in territorial behaviours with body size. Similarly, we used 15 cm bins to classify females as small (35–50 cm) and large (51+ cm), based on the size-distribution of females observed in mid-water schools in this study. Underwater visual size estimates of a subset of individuals were compared with size-estimates derived from focal videos of the same individuals using a scale reference (n = 20, see below). All individuals were correctly assigned to the respective size bins, and sizes were estimated within an error of ±5 cm.

The mean density of males and females and sex ratio was calculated in shelf and slope habitats by pooling point counts conducted in 2013 and 2014, as year did not have a statistically significant effect on mean density (see results). Sex ratio was calculated as the number of females as a proportion of total abundance in each habitat.

*Size-assortment* To study the distribution of small and large individuals (of males and females), we used generalised linear models (GLMs). Models were run separately for males and females. Count data from a total of 23 point-counts were used in the analysis. The density of males and females was modelled as a function of body size (large and small), year (2013, 2014), habitat (shelf, slope) and the interactions between habitat, size and year. We used negative-binomial glms to account for overdispersion in the data [48]. Only non-significant interaction terms (p < 0.05) were removed from the maximum model, to improve parameter interpretation [49, 50]. We used Likelihood ratio test for testing statistical significance of coefficients. Statistical hypothesis tests were not carried out for main effects involved in statistically significant interactions. Statistical analyses were performed with the statistical software R version 2.14.2 [51]. Negative-binomial glms were performed using lme4 [52]. Results were plotted using ggplot2 [53].

### Courtship tactics

#### Natural history observations

We observed the courtship behaviour of males and females, specifically the behaviour of female schools and territorial males. Where possible, video recordings were taken by placing GoPro Hero cameras at strategic locations on the reef. Each observed courtship behaviour was classified according to the location where it occurred (benthic or water column) and whether it was a pair courtship (between a single male and female) or a school courtship (single male and multiple females within a school, see results section for complete description). The size of female schools (number of females) was visually estimated underwater before the courtship survey (see below) and later corroborated from videos.

#### Distribution of male courtship tactics

The frequency of large and small males using pair and school-courtship tactics (see above) in the two habitats (slope, shelf) was estimated from focal individuals (n = 72) surveyed during an association-rate survey (see below). We used a contingency table to test if the courtship tactic used by large and small males was associated with the habitat they were found in. Since sample sizes in each cell of the 2 × 4 contingency table were low, we used a Fisher’s exact test to test the association [48].

### Costs and benefits of male ARTs

#### Benefits: association rates (potential mating opportunities)

Courtship took place either with females near the benthos (as in case of pair courtship) or with females within schools in the water column (school-courtship). We estimated association rate as the number of females a male courted per minute.

We measured association rates on the peak aggregation day (30th January) in 2014, with focal individual sampling. We sampled randomly identified males in each habitat and observed them for a period of 1 min (total n = 72). For each sampled individual we recorded the size of the male, the type of courtship it engaged in (pair or school) and the number of females it courted within 1 min. It was not possible to record these data blind because our study involved focal animals in the field.

We compared mean association rates of large and small males using pair and school courtship tactics on the shelf (n = 42) and slope (n = 30). Sampling with replacement was performed over 1000 iterations to produce 95% bootstrapped confidence intervals around the means. If the mean association rate of one population did not fall within the confidence intervals of the mean of the other, we considered the populations to be significantly different [48]. Bootstrapping was conducted using the R package, boot [54].

Mating rates are a challenge to measure in *P. areolatus* spawning aggregations because spawning presumably takes place at night or early morning, when surveys are difficult to conduct and because of the difficulties associated with measuring mating in externally fertilizing species. Very few researchers have observed gamete-release in *P. areolatus*, and gamete release has been reported only in male and female pairs after pair-courtship [42, 43, 45]. However, despite the difficulties associated with observing *P. areolatus* spawning, we observed two successful incidences of school-courtship culminating in gamete release. Both observations involved a single male with a group of females in a school. Since access to the number of females appears to differ considerably between courtship tactics, we assume that these would translate into differences in mating opportunities when spawning does take place. We therefore use association rates as a reasonable proxy for potential mating opportunities.

#### Costs: intra-sexual competition

To determine costs in terms of intra-sexual competition, we measured the proportion of time a male spent in aggressive interactions with other conspecific males. We used focal individual sampling (3 min) to obtain time activity budgets of males in shelf and slope habitats. Male focal individual samples (n = 65) were conducted on peak aggregation days in 2013 (10th February n = 14 slope and n = 14 shelf) and in 2014 (30th January, n = 18 slope, n = 19 shelf). Two observers swam from the northern to the southern edge of the aggregation site. Observers swam parallel to one another, one along the slope and the other along the shelf. During this swim, the observers randomly identified males in the two habitats and video recorded each individual for a 5-min period. Subsequent individuals were identified at a minimum distance of 5 m from the previous. Unique body marking were used to identify individuals in the videos. Individuals were followed at a minimum distance of four meters to minimise observer effects. We used a total sampling period of 5 min after initial observations. Males patrolled their entire territory within a minute on peak aggregation days; a sampling period of 5 min therefore provided us sufficient representation of an individual’s behavioural repertoire. During analyses the first 2 min of the recording were discarded to allow for focal individuals to acclimatize to our presence before we began scoring observations. Video data were recorded blind.

From the videos, we broadly classified behavioural states in males as:

*Rest* individual stationary in its territory, on top of, or under structures, maintaining its position with slow movements of its lateral and caudal fins.

We identified a sub-state within the ‘rest’ state called ‘perching’. Perching: individuals remain completely motionless, perched on top of structures in their territory with no fin movements.

*Rove* Any continuous swimming motion or ‘patrols’ made by the individual inside or outside its territory boundaries.

*Defence/aggression* individual chased an intruder from its territory, this state is different from a patrol in that it involved a directed high speed chase, involving flaring of dorsal fins and a colour change to a brown-marbled pattern, and was often followed by biting the intruder. Individuals that were stationary, but which displayed by flaring their dorsal fins and displaying the brown-marbled patterns were also included in this state.

*Courtship* Male courted a female (approaching with quivering motion of its body, followed by a display of his ventral side to the female, with or without body contact [42]).

A total of n = 32 focal individuals were sampled on the slope and n = 33 on the shelf. Separate models were used for each behavioural state. We modelled the effects of year (2013, 2014), habitat (shelf and slope) and the interaction between year × habitat on the binomial variable—total time spent in a particular behavioural state versus total time not spent in that state. Quasibinomial glms were performed to account for overdispersion [48] Only non-significant interaction terms (p < 0.05) were removed from the maximum model, to improve parameter interpretation [49, 50]. We used Likelihood ratio test for testing statistical significance of coefficients.

A summary of sampling tactics used for measuring different variables is provided in the “Appendix” section.

## Results

### Annual aggregation density

The estimated mean peak annual density of *P. areolatus* was 72.08 ± 27.46 fish per 1000 m^3^.

At the aggregation site, the mean density of fish on the slope (324 ± 130.58 fish per 1000 m^3^) was approximately six times higher than that on the shelf (59.4 ± 11.84 fish per 1000 m^3^).

### Male and female density distribution: size-assortment

**Fig. 1:**
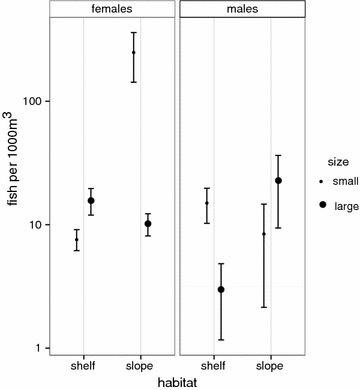
Size-assortment. Mean density ± SE (fish per 1000 m^3^) of large and small *P. areolatus* males (TL 40–55 cm, 56+ cm) and females (TL 35–50 cm, 51+ cm) in two habitats (shelf and slope) at the aggregation site in Lakshadweep. *Y*-*axis* plotted on log_10_ scale. Values averaged across 2 years 2013 and 2014 (n = 23 points)

Population sex ratios during peak aggregation days were highly skewed towards females on the slope (0.80), but were much more evenly balanced on the shelf (0.39). The density of small and large males (χ^2^ = 41.946, p < 0.0005) and females (χ^2^ = 24.413, p < 0.0005) changed substantially with habitat. The relative density of large males on the slope was approximately three times higher than small males. Conversely, the relative density of small males was 5 times higher than large males on the shelf (Fig. 1; Table 1). Large females were twice as abundant as small females on the shelf (Fig. 1; Table 1). In contrast, small females were 25 times more abundant than large females on the slope (Fig. 1; Table 1).

**Table 1:** Size assortment

Final models	Coefficients	Estimate	SE	Likelihood ratio test	
				χ^2^(df)	*p*
*Female.density* ~ *habitat* + *size* + *year* + *habitat* *×* *size*	Intercept (habitat: shelf, size: large, year: 2013)	1.629	0.395		
	Habitat: slope	−0.576	0.520		
Theta = 0.7819 ± 0.189	Size: small	−0.946	0.521		
df = 41	Year: 2014	0.023	0.365	0.003 (1)	0.949
Res.deviance = 51.04	Habitat: slope* size: small	4.381	0.732	24.413 (1)	<0.0005
*Male.density* ~ *habitat* + *size* + *year* + *habitat* *×* *size*	Intercept (habitat: shelf, size: large, year: 2013)	0.972	0.237		
	Habitat: slope	2.216	0.269		
Theta = 5.0346 ± 1.69	Size: small	1.800	0.268		
df = 41	Year: 2014	−0.115	0.167	0.470 (1)	0.49
Res.deviance = 52.552	Habitat: slope* size: small	−2.897	0.352	41.946 (1)	<0.0005

Negative-binomial GLM testing the relationship between male and female density with habitat (shelf and slope), body-size (large, small), year (2013, 2014) and the interactions between habitat, size and year at the aggregation site (n = 23 points). Maximum model with only the non-significant interaction terms removed to improve parameter interpretation. Statistical hypothesis testing carried out with likelihood ratio tests, except for main effects involved in statistically significant interactions

### Courtship tactics

#### Natural history observations

**Fig. 2:**
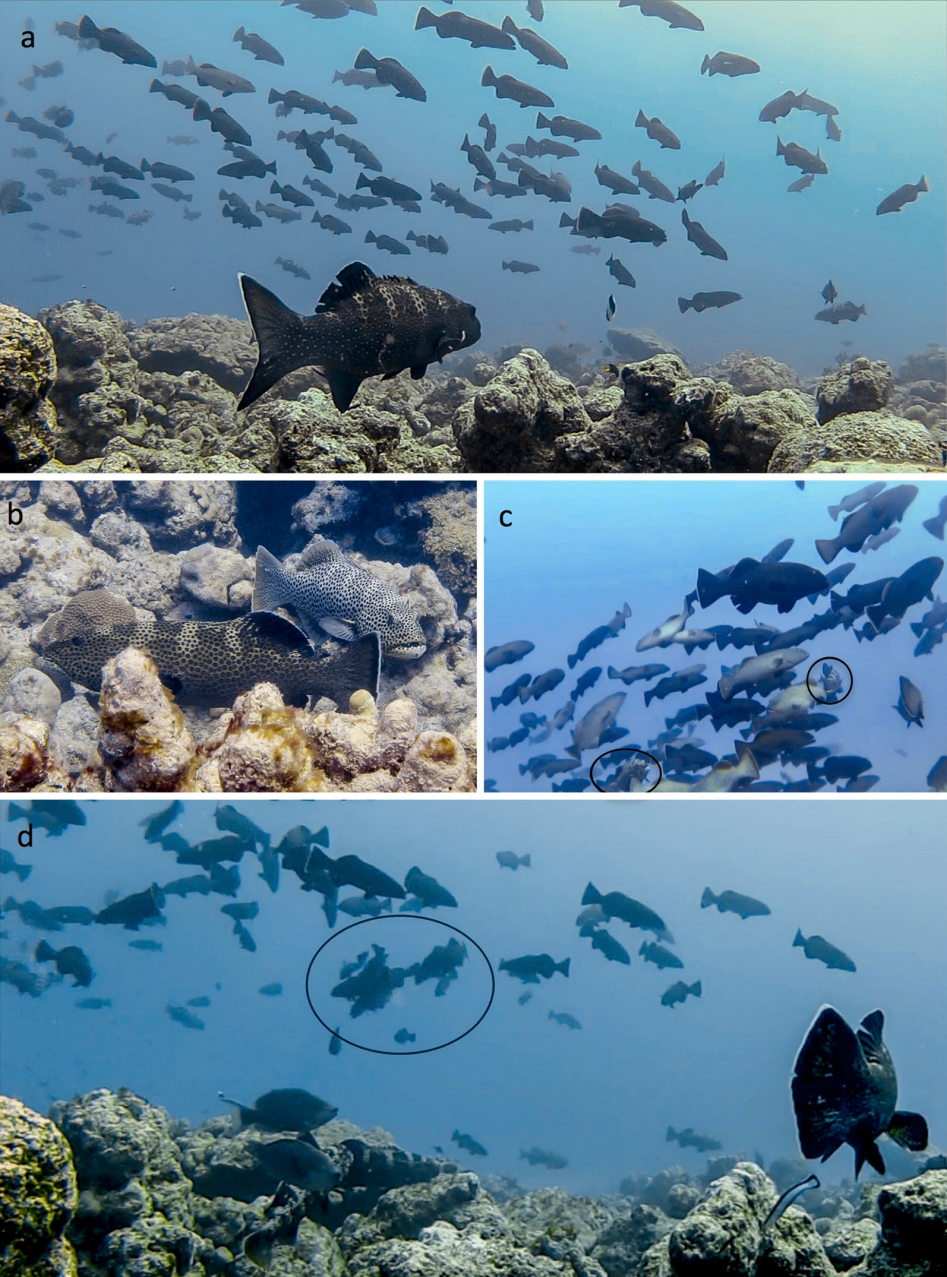
Courtship tactics. **a** Female schools: a school of small female squaretail groupers approaches the slope at the aggregation site. **b** Pair-courtship: a male squaretail grouper courts a female in its territory. This is a typical pair-courtship behaviour observed in *P. areolatus*. **c** School-courtship: two large territorial male squaretail groupers (*encircled*) making a foray into a female school >4 m above the benthos on the slope. **d** School-spawning: a novel school-spawning incident (*encircled*) observed between one large territorial male and a group of female squaretail groupers within a female school in the water column above the slope. This incident was captured on new moon eve, February 2013

We observed males arriving at the aggregation site up to 3 days prior to the new moon and establishing small, temporary territories (~5–10 m^−2^ area) on the reef slope and shelf. Both large and small males established territories at the aggregation site. These territories were maintained up to 2 days after peak spawning over the new moon phase. Females arrived at the aggregation area in large schools along the reef slope, a day prior to the peak aggregation day (Fig. 2a). We observed large schools of females (150–200 fish) moving around the core aggregation area and hovering in the mid-water column (i.e. stationary, with minimum movement of caudal and lateral fins) directly above the male territories. The female schools comprised of smaller individuals (<45 cm TL). Small females from these schools did not leave the school to disperse into male territories on the benthos. In contrast, large females (>45 cm TL) were observed roving independently along the benthos, or within male territories, but never as part of the schools (Additional file 1).

We recorded two distinct male courtship behaviours in this aggregation.

### Pair-courtship

Pair courtship took place between a territorial male and visiting female within the male’s territory. Pair courtship (approach, colour change, quivering motion, ventral side-display, quiver, and body contact, Fig. 2b) is often associated with pair-spawning; the latter involves a release of gametes by the pair in a spawning rush just above the male’s territory [42]. While we did not directly observe incidents of spawning after pair courtship in our study, this sequence has been previously documented in a study of *P. areolatus* aggregations [42, 43].

### School-courtship

School courtship behaviour involved males making regular ‘forays’ into female schools in the water column, above their territories (Fig. 2c). Males courted multiple females in the school during each foray, before returning rapidly to their territories. Courtship with females in the school was similar to that seen in pair courtship, with the difference that it took place in the mid-water column (3–4 m off the benthos) and simultaneously with multiple females.

We documented two distinct incidents of gamete release following this school courtship behaviour in the water column—one in 2013 (Fig. 2d) and another incident in 2014. Both events took place between one male and 4–5 females within a larger school. Females partaking in the spawning could be clearly identified based on their distended bellies. The incident involved an upward spawning rush within the school in the water column commonly seen in mass-spawning fish. Spawning took place >5 meters off the benthos (Additional file 2).

#### Distribution of male courtship tactics

Courtship tactics used by males varied with size (small and large) and habitat (shelf and slope, Fisher’s exact test p < 0.005). The school-courtship was more common among large males on the slope and less than a quarter of large males engaged in pair-courtship (Table 2). The frequency of small males using both school and pair-courtship on the slope was comparable and low (Table 2). The school-courtship tactic was completely absent on the shelf and all observed males (n = 30) engaged only in pair-courtship on the shelf (Table 2).

**Table 2:** Distribution of male courtship tactics

Habitat	Male size	Courtship tactic		Total sampled
		School^a^	Pair	
Slope	Large	20	4	23
	Small	11	8	19
Shelf	Large	0	4	4
	Small	0	26	26

The frequency of small (40–55 cm) and large (56+ cm) males involved in school or pair courtship in shelf and slope habitats (n = 72 males) at the aggregation site

### Costs and benefits of male ARTs

#### Benefits: association rates (potential mating opportunities)

**Fig. 3:**
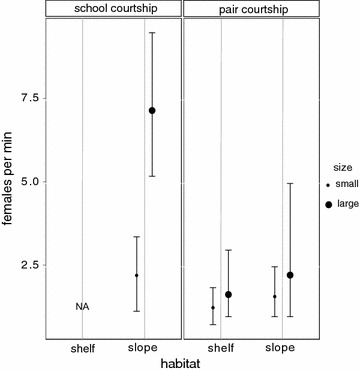
Male association rates. Mean association rates (number of females courted per minute) ±95% bootstrapped CIs of small (40–55 cm) and large (56+ cm) males (n = 72), using pair and school courtship tactics on the shelf and slope habitat at the aggregation site. The school-courtship tactic was not observed on the shelf despite the presence of female schools. Non-overlapping confidence intervals indicate significant differences in means

Large males courting schools on the slope, associated with seven times more females per unit time than small males on the slope, and three times more females per unit time than males engaged in pair courtship in both habitats (Fig. 3).

#### Costs: intra-sexual competition

**Fig. 4:**
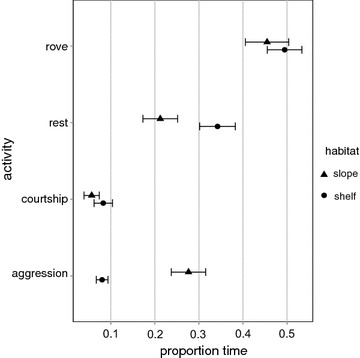
Male activity. Proportional time spent in an activity, by males (n = 71) on the slope and shelf at the aggregation site. *Closed circles* and *whiskers* represent mean ± SE values of shelf males, *triangles* represent values of slope males

The proportion of time spent in scored behavioural states did not change significantly between years (Fig. 4; Additional file 3). Time spent by males in aggressive behaviour was considerably higher (up to four times) on the slope than the shelf (χ^2^ = −845.900, p < 0.0005). Conversely, males spent twice as much time resting on the shelf than slope (χ^2^ = −347.97, p < 0.0005) Time spent in courtship and roving behaviours did not vary between habitats (Fig. 4; Additional file 3).

## Discussion

Species often employ unusual reproductive tactics while mating at very high densities [1, 2, 4]. Fish spawning aggregations can provide unique opportunities to study such unusual, density-dependent mating tactics at high population densities. However, our understanding of natural mating systems of many commercially exploited, aggregating fish species is often obscured by the high anthropogenic pressures their populations sustain. At the time of our observations, the *Plectropomus areolatus* aggregation in Bitra represented one of the few unfished spawning aggregations of a large-bodied marine fish, with the highest recorded densities for this species across the Indo-Pacific (*Palau* [42]; *Indonesia* [43]; *Western Solomon islands,* [55]; *Papua New Guinea* [56]; *Pohnpei* [57]). At these unfished densities, we observed two peculiarities in the *P. areolatus* mating system compared to other locations. Firstly, there appeared to be an inverse size-assortment between males and females at the aggregation site in Bitra. Secondly, we observed two distinct male courtship tactics: pair courtship and school courtship—the latter appears to be a novel courtship tactic in this population. Perhaps more interesting than these two distinct courtship tactics were the opportunistic observations of spawning after school courtship, suggesting that the type of courtship tactic (pair or school) may lead to two distinct and alternative reproductive tactics. Of the two ARTs, pair-spawning, is a commonly reported tactic in *P. areoaltus* and is associated with pair-courtship [40, 42, 43]. In contrast, school-spawning is a unique tactic in this species, which we describe for the first time in the Bitra spawning aggregation. Given the extremely high densities of individuals observed in this spawning aggregation, we suggest that the unique school-spawning tactic in *P. areolatus* is likely seen only in very high-density populations. This could explain why school-spawning has been previously unreported from studies across the Indo-Pacific. Opportunistic studies from unfished populations such as these can thus provide important baseline information on unique mating strategies of species at naturally high densities.

## Inverse size-assortment

Overall the *P. areolatus* aggregation attained its highest density on the slope, as described at other locations across the Indo-Pacific [55–58]. Perhaps the most intriguing characteristic of the Bitra spawning aggregation is the inverse size-assortment of males and females, contrary to positive size-assortment, which is commonly seen in fish [16]. At first glance, this inverse-size assortment appears counter-intuitive. If female distribution were strongly influenced by the distribution of males alone, we would expect large females to be relatively more abundant in the high-density slope habitat where large males were present, which was contrary to our observation. Typically, females choose larger males as mates for their superior quality and quantity of gametes [19, 59]. However, size-assortment in individuals can be weak when the costs of mating with a larger partner (asymmetrical exploitation, intra-sexual competition) are not outweighed by size-related mating advantages [60], or simply because body-size is not a male trait that directly affects fitness [61–63]. Alternatively, females may be indifferent towards male size [64] if they select external environmental cues like predation pressure, or site quality to spawn [25, 30, 65]. Whether female distribution were a consequence of mate choice, cryptic competition and/or a choice for certain habitat characteristics would require careful manipulative experiments, which were beyond the scope of this opportunistic, observational study. Irrespective of the mechanisms however, it appears that female behaviour may have a strong influence on male distribution in this aggregation.

We observed large males preferentially courting small females within schools on the slope, despite the presence of larger females on the slope. Female schools have been reported at other locations of *P. areolatus* aggregations across the Indo-Pacific, but tend to be much smaller in number (15–45 individuals per school [42, 43]). In comparison, the female schools we observed were an order of magnitude larger (>150 individuals) and unique only to Bitra atoll in Lakshadweep (RA, personal observation, [66]). While at this juncture we can only speculate on the mechanisms underlying this inverse-size assortment, it appears to be clearly unique to the high-density *P. areolatus* spawning aggregation in Bitra and is currently undocumented in other aggregations.

## A unique mating tactic?

An exciting observation in this study is the multiple incidents of a unique mating tactic, school-spawning. The two incidents of school-spawning were remarkably similar in nature, and unique to other tactics in two ways. For one, females within schools simultaneously released gametes as a cohesive unit, and did not disperse into male territories to individually pair spawn after being courted by males [42]. For another, the school-spawning tactic differed from traditional observations of ‘group spawning’ because it involved a single male and multiple females partaking in an upward spawning rush, and not a single female and multiple males, which traditionally defines ‘group spawning’ [67]. It is likely that the school-spawning tactic may be a variation of group spawning, in which multiple males eventually join and simultaneously spawn within the school as seen in mass-spawning fish [68]. With these limited observations, we cannot preclude the possibility that school-courtship may also lead to pair-spawning or mass-spawning, as has been traditionally explained [42]. However, our opportunistic observations clearly suggest that in rare circumstances school-courtship may lead to a unique school-spawning tactic, likely only in very high density *P. areolatus* aggregations.

## Male ARTs: patterns and processes

Alternative reproductive tactics are observed in mating populations, when individuals adopt distinct and alternative ways to maximize their reproductive benefits in the context of intra-sexual reproductive competition [69]. Unpredictability in partner availability, competition and predation risk, often selects for flexible and simultaneous ARTs, which are common in fish [69]. The two distinct ARTs in the high density *P. areolatus* aggregation appeared to be conditional upon potential mating opportunities and male competitive abilities. The slope habitat appeared to be the preferred habitat at the aggregation site—and this is likely associated with high mate encounter rates [15, 59] or potential mating opportunities generated by the movement of female schools. In addition, inter-specific competition was found to be four times higher among males on the slope than shelf. Large males had a clear size-related competitive advantage [70, 71] over their smaller counterparts and dominated the slope habitat. The largest males in this population were nearly 1.5 times longer than the smallest males. Further, on the high-density slope, large males engaged in school courtship much more frequently than pair courtship. While it is true that school courtship afforded seven times higher potential mating opportunities to the large males than pair courtship, it appeared to be a highly risky tactic because males had to leave their territories unattended during school forays. Despite higher levels of intra-sexual competition however, it appears that the benefits large males potentially gained by spawning within female schools likely offset these costs, selecting for this unique and costly mating tactic by large males in the high-density slope habitat.

Smaller males in contrast were significantly disadvantaged on the reef slope. We observed large males aggressively chasing away and injuring smaller competitors that attempted school-courtship. With high intra-sexual competition and no significant gains in potential mating opportunities, using the school-courtship tactic offered few benefits for small males on the slope. However, pair-courtship yielded similar potential mating opportunities in both habitats for small males, and these were associated with significantly lower levels of intra-sexual competition especially on the shelf. Taken together, males in this high-density spawning aggregation appear to adopt two distinct and flexible ARTs: a ‘school-courtship tactic’, which is a high-cost-high-benefit tactic associated with school-spawning, and a ‘pair-courtship tactic’, which is a low-cost-low-benefit tactic associated with pair-spawning.

## Conclusion

To our knowledge our observations of two distinct courtship tactics and inverse size-assortment is the first reported for *P. areolatus*. Crucially these properties only occur in Lakshadweep where the aggregation was unfished and aggregating densities were much high than those reported in the rest of the Indo-Pacific. Our study therefore poses an important conservation question; if *P. areolatus* populations in Bitra are exposed to fishing pressures, could it lead to a loss of the rare inverse-size assortment and unique school-courtship tactics from *P. areolatus* spawning aggregation? Commercial fishing of groupers at the aggregation site in Bitra has recently commenced (2013). Our most recent density census from 2015 and 2016 show that the peak aggregation density in January has declined by an alarming 50% compared to 2013. With an offtake pressure estimated at 12–15 tonnes of fish in 2015 (RA, RK unpublished data), the declining density is likely a result of this newly emerging commercial reef fishery. While the impact of the fishery on the unique *P. areolatus* mating system still remains to be evaluated, no female-schools or school-courtship were observed during surveys in 2016. This study raises several questions about the evolution and maintenance of this unusual ‘school spawning’ tactic in high-density *P. areolatus* aggregations. However, we fear that this opportunity may be lost due to the fast declining population densities of the Bitra aggregation. Opportunistic studies from unfished populations such as these can thus provide important baseline information on unique mating strategies of species at naturally high, unfished densities.

## Appendix

See Table 3.

**Table 3:** A summary of sampling techniques and sample sizes used for estimating variables

Variable	Sampling technique	Sample size and factors	Tests performed
Annual peak aggregation density	UVC permanent belt transects (transect volume = 50*10*5 m^3^)	N = 10 transects Year (2013, 2014) Habitat (slope, shelf)	–
Size-assortment: male and female density distribution	UVC points (point volume = π*5*5 m^3^)	N = 23 points Year (2013, 2014), Habitat (shelf, slope) Size (large, small)	Negative-binomial glm, likelihood ratio tests
Frequency of male courtship tactics	Focal individual sampling, 1 min underwater observations	N = 72 (2014) focal males Habitat (shelf, slope)	2 × 2 Contingency table, Fisher’s exact test
Benefits: association rates (potential mating opportunities)	Focal individual sampling, 1 min underwater observations	N = 72 (2014) focal males Habitat (shelf, slope)	95% Bootstrapped confidence intervals
Costs: intra-sexual competition	Focal individual sampling, Activity budgets, 5 min videos	N = 65 (2014) focal male videos Habitat (shelf, slope)	Quasibinomial glms, likelihood ratio tests

## Additional files

**Additional file 1.** Female schools: schools of gravid females roving at the aggregation site. Male squaretail groupers (brown-marbled colouration) are seen making forays into the school and courting multiple females.

**Additional file 2.** School-spawning incident: a school-spawning incident observed in 2013. A male squaretail grouper from the slope males leaves his territory to make a foray into the school. Male is seen courting multiple females in the school. This school-courtship is followed by a sudden upward spawning rush between the male and 4–5 females from the school, proceeded by the release of gametes.

**Additional file 3.** Male activity. Quasibinomial GLMs modelling the effect of habitat (slope, shelf), year (2013, 2014) and their interaction on the total time spent by males (n = 65) in an activity (aggression, courtship, rest, rove) versus time not spent in that activity. Maximum model with only the non-significant interaction are terms removed to improve parameter interpretation. Statistical hypothesis testing of coefficients carried out with likelihood ratio tests.

## Abbreviation

ART: alternative reproductive tactics
