# Supplementary material for: Alternative reproductive tactics and inverse size-assortment in a high-density fish spawning aggregation
Source: BMC Ecol. 2017 Feb 28;17:10. doi: 10.1186/s12898-017-0120-5 (PMC5331645; doi:10.1186/s12898-017-0120-5)
Supplement: Supplementary file 3 — Additional file 3. Male activity. Quasibinomial GLMs modelling the effect of habitat (slope, shelf), year (2013, 2014) and their interaction on the total time spent by males (n = 65) in an activity (aggression, courtship, rest, rove) versus time not spent in that activity. Maximum model with only the non-significant interaction are terms removed to improve parameter interpretation. Statistical hypothesis testing of coefficients carried out with likelihood ratio tests. [file 12898_2017_120_MOESM3_ESM.docx]

| **final models** | | **coefficients** | **estimate** | | **SE** | | **Likelihood ratio test** | | | |  |
| --- | --- | --- | --- | --- | --- | --- | --- | --- | --- | --- | --- |
|  |  |  |  |  |  |  | **χ^2^(df)** | | ***P*** | |  |
| **1. aggression ~ habitat + year** |  | | |  | |  | |  | |  | |
| dispersion parameter =33.43 | **intercept** (habitat: shelf, year: 2013) | | | -2.644 | | 0.311 | | -882.47(2) | | <0.0005 | |
| df =62 | **habitat**: slope | | | 1.434 | | 0.310 | | -845.9 (1) | | <0.0005 | |
| res.deviance = 2082 | **year**: 2014 | | | 0.355 | | 0.274 | | -56.84 (1) | | 0.196 | |
|  |  | | |  | |  | |  | |  | |
| **2. courtship ~ habitat + year** |  | | |  | |  | |  | |  | |
| dispersion parameter =37.63 | **intercept** (habitat: shelf, year: 2013) | | | -2.460 | | 0.355 | | -38.882(2) | | 0.596 | |
| df =62 | **habitat**: slope | | | -0.415 | | 0.424 | | -36.51(1) | | 0.324 | |
| res.deviance = 1926.6 | **year**: 2014 | | | 0.087 | | 0.419 | | -1.61(1) | | 0.836 | |
|  |  | | |  | |  | |  | |  | |
| **3. rest ~ habitat + year** |  | | |  | |  | |  | |  | |
| dispersion parameter =52.438 | **intercept** (habitat: shelf, year: 2013) | | | -0.281 | | 0.232 | | -596.92(2) | | <0.0005 | |
| df =62 | **habitat**: slope | | | -0.729 | | 0.286 | | -347.97(1) | | <0.0005 | |
| res.deviance = 3526.1 | **year**: 2014 | | | -0.658 | | 0.287 | | -282.32(1) | | 0.020 | |
|  |  | | |  | |  | |  | |  | |
| **4. rove ~ habitat + year** |  | | |  | |  | |  | |  | |
| dispersion parameter =54.489 | **intercept** (habitat: shelf, year: 2013) | | | -0.216 | | 0.226 | | -81.09(2) | | 0.475 | |
| df =62 | **habitat**: slope | | | -0.062 | | 0.255 | | -76.301(1) | | 0.236 | |
| res.deviance = 3857 | **year**: 2014 | | | 0.302 | | 0.255 | | -3.234(1) | | 0.807 | |

S3 Male activity

Quasibinomial GLMs modelling the effect of habitat (slope, shelf), year (2013,2014) and their interaction on the total time spent by males (n=65) in an activity (aggression, courtship, rest, rove) versus time not spent in that activity. Maximum model with only the non-significant interaction are terms removed to improve parameter interpretation. Statistical hypothesis testing of coefficients carried out with likelihood ratio tests.
